# Concomitant Guillain Barre Syndrome and Transverse Myelitis as Initial Neuropsychiatric Manifestation in a Case of Lupus: A Diagnostic Quandary

**DOI:** 10.1155/2016/5827860

**Published:** 2016-05-08

**Authors:** Anshuman Srivastava, Bijit Kumar Kundu, Diwakar Kumar Singh

**Affiliations:** ^1^Department of Medicine, PGIMER and Dr. RML Hospital, New Delhi 110001, India; ^2^Rheumatology Clinic, Department of Medicine, PGIMER and Dr. RML Hospital, New Delhi 110001, India

## Abstract

Neuropsychiatric manifestations of systemic lupus erythematosus are varied. Presently nineteen in number, they are classified as whether affecting the central or the peripheral compartments of the nervous system. Its diagnosis however remains difficult, more so when two or more of the syndromes are found concomitantly in the same patient and when they occur in absence of the more classical rash, serositis, and haematological manifestations. We present a case of lupus where myelopathy as well as demyelination existed simultaneously as the initial neurologic manifestation.

## 1. Introduction

The neuropsychiatric manifestations of systemic lupus erythematosus (SLE), known commonly as neuropsychiatric SLE (NPSLE), include 19 different entities divided into central or peripheral affections which may be attributable directly to systemic lupus erythematosus (SLE) or associated with it in increasing frequencies. These do not correlate with systemic disease activity and can occur in combination. Transverse myelitis (TM) is a devastating manifestation, can occur at any time in the course of the disease, and involves small segments or the whole of the spinal cord anywhere from the medulla to the conus. Ascending paralysis similar to Guillain Barre Syndrome (GBS) has also been described. These are rare and along with chorea make up less than 3% of the cases of NPSLE, a fact which attests to the rarity of these manifestations. Compounding this rare scenario is the fact that the clinical distinction of demyelination and myelopathy can be difficult in the initial stages, both having overlapping features. Radiological and electrophysiological evidence can be incongruent to the clinical features as well as to each other, adding to the confusion already present. We present such a case where a 35-year-old female had features of both leading to diagnostic quandary.

## 2. Case Presentation

**Figure 1:**
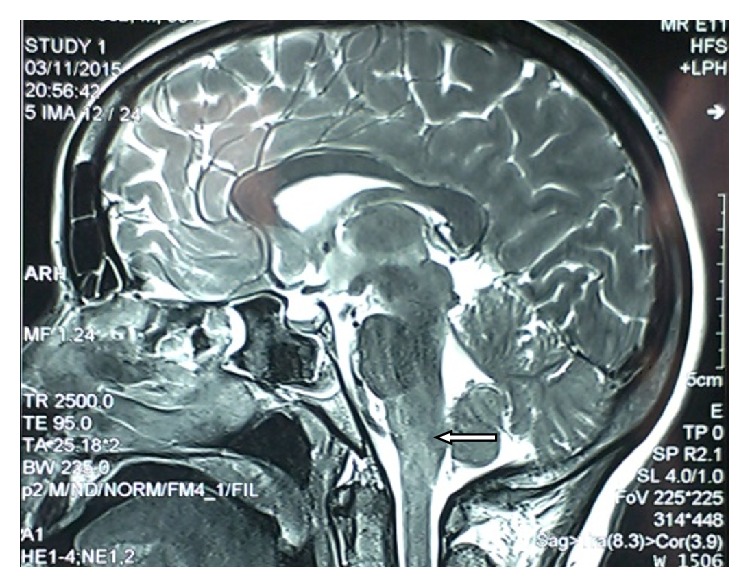
MRI of the brain in sagittal section showing hyperintensities at the cervicomedullary junction (white arrow).

**Figure 2:**
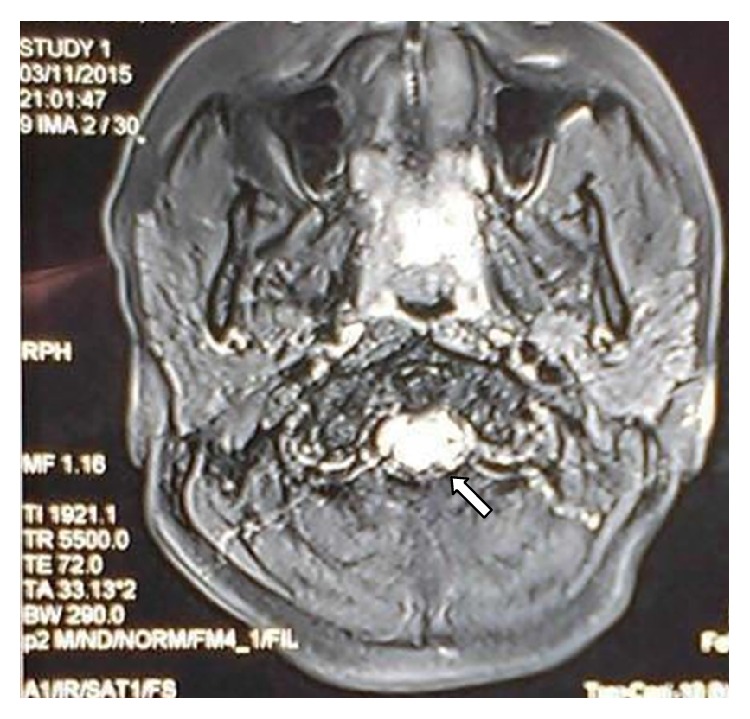
The same hyperintensity shown in the transverse section (white arrow).

A 30-year-old lady presented with complaints of progressive ascending weakness of both lower limbs for a week for which a diagnosis of GBS was made elsewhere. After few days she also developed incontinence of urine and stools. A Magnetic Resonance Imaging (MRI) done then revealed myelitis of the spine at junction of the medulla and cervical spine. Owing to the diagnostic quandary, she was referred to our centre for further evaluation. Review of her history revealed a course consistent with the diagnosis of GBS but lack of any prodrome or viral illness prior to the onset of the weakness. Past history revealed occurrence of pain with minimal swelling affecting the small joints of hands, wrists, knees, and ankles, associated with Raynaud's phenomenon. These symptoms had been occurring for 2-3 months every winter for the last three years and always subsided with onset of summers. Only this time, the resolution of symptoms was not complete. She had been treated intermittently with nonsteroidal anti-inflammatory drugs (NSAIDs) and low dose corticosteroids by her general practitioner (GP). She had two healthy children, was a teetotaler, never suffered from tuberculosis, was not a known diabetic or hypertensive, and did not give any history of abortions. Family history was insignificant. General examination was unremarkable except for pallor. Central nervous system (CNS) examination revealed a conscious oriented lady with flaccid quadriparesis, areflexia, and unresponsive plantar reflexes. Cardiovascular, respiratory, and abdominal examinations were normal. Musculoskeletal examination revealed minimal to mild synovitis in few metacarpophalangeal (MCP) and interphalangeal (IP) joints of both hands and wrists. Laboratory investigations revealed haemoglobin (Hb) 9.2 g/dL, total leucocyte count (TLC) 7800/mm^3^, total platelet count of 120000/mm^3^, a normocytic normochromic blood picture, serum sodium (Na^+^) 142 mEq/dL, serum potassium (K^+^) of 4.6 mEq/dL, and normal liver and kidney function tests. Chest radiograph was reported to be normal. Serology for Human Immunodeficiency Viruses (HIV) types 1 and 2, Hepatitis B Virus Surface Antigen (HBsAg), Hepatitis C Virus (HCV), Herpes Simplex Virus (HSV) types 1 and 2, and Cytomegalovirus (CMV) was negative. Cerebrospinal fluid (CSF) analysis showed albuminocytological dissociation with sugar 85 mg/dL and proteins 110 mg/dL and no cells. MRI of brain and spinal cord reviewed showed hyperintensities in T2 weighted images in cervicomedullary junction (Figures 1 and 2). Nerve conduction study (NCS) showed pure motor involvement with increased distal latencies, decreased conduction velocities, normal amplitude, and absent F-waves with no affection of sensory nerves, features which in the background of rapidly progressive ascending areflexic quadriplegia suggested the diagnosis of the Acute Inflammatory Demyelinating Polyneuropathy (AIDP) subtype of GBS. Anti-Nuclear Antibody (ANA) was positive in 1 : 640 dilution and homogeneous in pattern by indirect immunofluorescence (IIF), and anti-smith antibody (anti-Sm) was positive with intensity of 65 by EUROLine scan. Levels of complement component C3 were low (76.2 mg/dL, normal 90–180 mg/dL) while component C4 levels were towards the lower limits of normal (10.5 mg/dL, normal 10–40 mg/dL). Direct Coombs test (DCT), rheumatoid factor (RF), and cyclic citrullinated peptide antibodies (anti-CCP) were negative. A provisional diagnosis of SLE with CNS involvement was kept in view of arthritis, CNS involvement, positive ANA, and low complements. Treatment was instituted immediately with intravenous methyl prednisolone 1 g once a day for three days followed by oral prednisolone at 1 mg/kg body weight daily, hydroxychloroquine 200 mg twice a day, and cyclophosphamide 750 mg infusion. Her neurologic status improved and a repeat neurological examination after 3 days showed improved power of 2/5 in both upper limbs. After two weeks her muscle power improved to 2/5 around hip joints and 3/5 around shoulders, elbows, knees, and ankle joints. Her bladder and bowel functions also improved and she could be decatheterised. Subsequent MRI screen of spine repeated after 3 weeks was normal.

The patient was put on monthly pulses of cyclophosphamide, and steroids were continued at 1 mg/kg body weight for 6 weeks and then tapered by 10% of the dose every two weeks. After 12 weeks, she had grade 4− (4 minus) muscle power around hips. Power around shoulders, elbows, wrists, knees, and ankles was grade 4. Grip was normal. She is able to carry out most of her activities of daily living.

## 3. Discussion

The prevalence as well as the prognosis of neuropsychiatric events in SLE is highly variable with almost a third being directly attributable to it. These have a negative effect on the health related quality of life [1]. The American College of Rheumatology (ACR) in 1999 defined 19 neuropsychiatric syndromes which occur in lupus. They can be classified into those affecting the central or the peripheral nervous systems [2] and are thought to be due to the effects of vascular abnormalities, different autoantibodies, and mediators of inflammation.

The 1997 ACR criteria included only seizures and organic brain syndrome (in absence of offending drugs or known metabolic derangements like uraemia, ketoacidosis, or electrolyte imbalance) in its criteria. The neurological manifestations of lupus in Systemic Lupus International Collaboration Clinics (SLICC) criteria include seizures, psychosis, mononeuritis multiplex (in the absence of other known causes such as primary vasculitis), myelitis, peripheral or cranial neuropathy (in the absence of other known causes such as primary vasculitis, infection, and diabetes mellitus), and acute confusional state (in the absence of other causes, including toxic/metabolic, uraemia, and drugs). The SLICC criteria containing many manifestations with less stringent timeframes aid in earlier diagnosis of NPSLE [3, 4].

Interestingly the commonest manifestations of NPSLE, namely, headache, psychosis, mood disorders, anxiety, and cognitive dysfunction, are the least specific. Still confounding the scenario is the fact that these syndromes have no correlation with disease activity and can occur at any stage of the disease, and in combination with other neurologic manifestations, thus making diagnosis difficult and liable to be missed and hence delaying treatment.

Among the rarer manifestations of SLE are an acute ascending demyelination akin to the classical Guillain Barre Syndrome (GBS) and transverse myelitis (TM) which are taught in medical schools. Though classical GBS and TM due to nonlupus causes are seen fairly commonly in medical school setting, those occurring as a result of lupus are seen in less than 3% of patients of lupus. However in lupus, TM is catastrophic, can occur at any time of the disease, and can involve small segments or entire spinal cord from medulla to conus. Though different syndromes in lupus are known to coexist or overlap, overlap of TM and GBS in the same patient of lupus could not be found in literature. However, coexistent TM and GBS have been reported in mycoplasma pneumonia in adolescents [5], in mumps [6], and after anti-Tumour Necrosis Factor (TNF) therapy [7].

GBS and TM can be difficult in absence of the classical rash of SLE to be attributed to as being due to SLE. Also watertight distinction between these two clinicopathologic entities may not be possible especially in the initial stages of manifestation because of overlapping clinical features and incongruence between clinical and radiological features. They are thus usually inferred to be due to causes other than SLE. Distinguishing even nonlupus TM and GBS assumes importance in view of the fact that GBS is usually treated with intravenous immunoglobulin (IVIg) or plasmapheresis, while steroids have no role. To elicit the cause of GBS and TM as SLE is of paramount importance because treatment of the same would include cytotoxic agent like cyclophosphamide and pulse steroids and because of the fact that, in spite of early diagnosis and treatment, only half of the patients show improvement [8].

Our patient had clinical findings suggestive of GBS with the nerve conduction study confirming the diagnosis. However MRI of brain and spine showed features of myelitis too. Retrospectively the whole clinical scenario could be explained by myelitis with spinal shock except for the gradual onset and the NCS findings. Thus our patient was a diagnostic dilemma with varied opinions and no unifying one till the diagnosis of NPSLE was made. Nonetheless immediate institution of pulse steroids and cytotoxic treatment led to improvement in the patient.

Test for anti-phospholipid antibodies (APLA) could not be done in our patient due to affordability issues and constitutes a limitation in our case. Anti-phospholipid syndromes (APS) either primary or secondary can be associated with various neurologic syndromes including transverse myelitis and GBS. The revised Sapporo or Sydney criteria [9] mandate fulfilment of at least one criteria clinical criterion, that is, vascular thrombosis or pregnancy related morbidity, and at least one laboratory criterion in order to make a diagnosis of APS. However our patient does not fulfil any of the two clinical criteria and thus APS could be presumed to be negative, albeit indirectly.

Behcet's disease (BD), sarcoidosis, vitamin B12 deficiency, vitamin D deficiency, and multiple sclerosis (MS) can share some of the clinical features of our case. However, they can be easily ruled out by detailed history, clinical examination, and basic investigations. BD can cause parenchymal lesions in brainstem as well as nonparenchymal lesions like aseptic meningitis, intracranial hypertension, cranial neuropathies or dural sinus thrombosis, arterial dissection, occlusion, and aneurysm [10], while sarcoidosis can cause cranial neuropathy, aseptic meningitis, mass lesions, encephalopathy, vasculopathy, seizures, hypothalamic-pituitary disorders, hydrocephalus, myelopathy, peripheral neuropathy, and myopathy and is usually suspected in a known case of sarcoidosis [11]. While absence of oral ulcers, a sine qua non for diagnosis of BD, along with high titre ANA in homogeneous pattern and albuminocytological dissociation in CSF rules out BD, the latter two along with no hilar lymphadenopathy on chest radiograph rule out sarcoidosis. Vitamin B12 deficiency can manifest as encephalopathy, myelopathy, peripheral neuropathy, and optic neuropathy. Reflexes are usually absent and weakness can be present [12]. However, the course is more prolonged, sensations are affected, and blood picture usually shows abnormalities like megaloblastic anaemia and hypersegmented neutrophils, features which were absent in our patient. Vitamin D deficiency can have proximal muscle weakness with normal sensations but can be easily distinguished by tender muscles, preserved reflexes, and normal CSF and NCS findings. Though MS presents in the same age group as our patient and is commoner in females, it is disseminated in time and space, spares the peripheral nervous system, and commonly affects the optic and sensory nerves, and associated systemic illness is not found, features which were absent in our patient [13].

It is our contention that, in any case of GBS and/or TM occurring singly or concomitantly, it is vital to keep the differential diagnosis of SLE especially in a lady of child bearing age even in the absence of any rash or other pointers to its diagnosis, more so if there is any incongruity between clinical, radiological, and electrophysiological findings. Sensitivity to and consideration of an autoimmune aetiology lead to prompt evaluation, early diagnosis, and early institution of treatment thus helping in improving prognosis.

## References

[1] Hanly J. G., Lahita R. G. (2011). The nervous system and lupus. *Systemic Lupus Erythematosus*.

[2] American College of Rheumatology Ad Hoc Committee on Neuropsychiatric Lupus Nomenclature (1999). The ACR nomenclature and case definitions for neuropsychiatric lupus syndromes. *Arthritis & Rheumatology*.

[3] Amezcua-Guerra L. M., Higuera-Ortiz V., Arteaga-García U., Gallegos-Nava S., Hübbe-Tena C. (2015). Performance of the 2012 Systemic Lupus International Collaborating Clinics and the 1997 American College of Rheumatology classification criteria for systemic lupus erythematosus in a real-life scenario. *Arthritis Care and Research*.

[4] Ines L. S., Silva C., Galindo M. (2015). Classification of systemic lupus erythematosus: systemic lupus international collaborating clinics versus american college of rheumatology criteria. A comparative study of 2,055 patients from a real-life, international systemic lupus erythematosus cohort. *Arthritis Care & Research*.

[5] Topcu Y., Bayram E., Karaoglu P., Yis U., Guleryuz H., Kurul S. H. (2013). Coexistence of myositis, transverse myelitis, and Guillain Barré syndrome following *Mycoplasma pneumoniae* infection in an adolescent. *Journal of Pediatric Neurosciences*.

[6] Bajaj N. P. S., Rose P., Clifford-Jones R., Hughes P. J. (2001). Acute transverse myelitis and Guillain-Barré overlap syndrome with serological evidence for mumps viraemia. *Acta Neurologica Scandinavica*.

[7] Ugurlu S., Saygin C., Uzunaslan D., Tascilar K., Ince B., Saip S. (2014). Concomitant transverse myelitis and guillain-barre syndrome after adalimumab therapy in a case with ankylosing spondylitis. *Annals of the Rheumatic Diseases*.

[8] Hochberg M. C., Silman A. J., Smolen J. S., Weinblatt M. E., Weisman M. H. (2015). *Rheumatology*.

[9] Miyakis S., Lockshin M. D., Atsumi T. (2006). International consensus statement on an update of the classification criteria for definite antiphospholipid syndrome (APS). *Journal of Thrombosis and Haemostasis*.

[10] Shahien R., Bowirat A. (2010). Neuro Behcet’s disease: a report of 16 patients. *Journal of Neuropsychiatric Disease and Treatment*.

[11] Kumholz A., Stern B. J. (2014). Neurologic manifestations of sarcoidosis. *Handbook of Clinical Neurology*.

[12] Scherer K. (2003). Neurologic manifestations of vitamin B_12_ deficiency. *The New England Journal of Medicine*.

[13] Hauser S. L., Goodin D. S., Casper D. L., Fauci A. S., Hauser S. L., Longo D. L., Jameson J. L., Loscalzo J. (2015). Multiple sclerosis and other demyelinating diseases. *Harrison's Principles of Internal Medicine*.

